# Ultrasonography, magnetic resonance imaging, radiography, and clinical assessment of inflammatory and destructive changes in fingers and toes of patients with psoriatic arthritis

**DOI:** 10.1186/ar2327

**Published:** 2007-11-14

**Authors:** Charlotte Wiell, Marcin Szkudlarek, Maria Hasselquist, Jakob M Møller, Aage Vestergaard, Jesper Nørregaard, Lene Terslev, Mikkel Østergaard

**Affiliations:** 1 Department of Rheumatology, University of Copenhagen Hvidovre Hospital, Kettegaard Allé 30, 2650 Hvidovre, Denmark; 2 Department of Diagnostic Radiology, University of Copenhagen Herlev Hospital, Herlev Ringvej 75, 2730 Herlev, Denmark; 3 Department of Radiology, University of Copenhagen Hvidovre Hospital, Kettegaard Allé 30, 2650 Hvidovre, Denmark; 4 Department of Rheumatology, University of Copenhagen Nordsjællands Hørsholm Hospital, Usserød Kongevej 102, 2970 Hørsholm, Denmark; 5 Department of Rheumatology, University of Copenhagen Herlev Hospital, Herlev Ringvej 75, 2730 Herlev, Denmark

## Abstract

The aim of the present study was to assess ultrasonography (US) for the detection of inflammatory and destructive changes in finger and toe joints, tendons, and entheses in patients with psoriasis-associated arthritis (PsA) by comparison with magnetic resonance imaging (MRI), projection radiography (x-ray), and clinical findings. Fifteen patients with PsA, 5 with rheumatoid arthritis (RA), and 5 healthy control persons were examined by means of US, contrast-enhanced MRI, x-ray, and clinical assessment. Each joint of the 2nd–5th finger (metacarpophalangeal joints, proximal interphalangeal [PIP] joints, and distal interphalangeal [DIP] joints) and 1st–5th metatarsophalangeal joints of both hands and feet were assessed with US for the presence of synovitis, bone erosions, bone proliferations, and capsular/extracapsular power Doppler signal (only in the PIP joints). The 2nd–5th flexor and extensor tendons of the fingers were assessed for the presence of insertional changes and tenosynovitis. One hand was assessed by means of MRI for the aforementioned changes. X-rays of both hands and feet were assessed for bone erosions and proliferations. US was repeated in 8 persons by another ultrasonographer. US and MRI were more sensitive to inflammatory and destructive changes than x-ray and clinical examination, and US showed a good interobserver agreement for bone changes (median 96% absolute agreement) and lower interobserver agreement for inflammatory changes (median 92% absolute agreement). A high absolute agreement (85% to 100%) for all destructive changes and a more moderate absolute agreement (73% to 100%) for the inflammatory pathologies were found between US and MRI. US detected a higher frequency of DIP joint changes in the PsA patients compared with RA patients. In particular, bone changes were found exclusively in PsA DIP joints. Furthermore, bone proliferations were more common and tenosynovitis was less frequent in PsA than RA. For other pathologies, no disease-specific pattern was observed. US and MRI have major potential for improved examination of joints, tendons, and entheses in fingers and toes of patients with PsA.

## Introduction

Arthritis in small joints is common in psoriasis-associated arthritis (PsA), and the clinical distinction from rheumatoid arthritis (RA) can be difficult [1]. Improved therapy options and knowledge of the importance of early initiation of aggressive treatments to optimize long-term outcome in patients [2-5] have led to an increasing focus on developing new sensitive diagnostic and monitoring tools. Imaging modalities such as ultrasonography (US) and magnetic resonance imaging (MRI) appear promising. MRI can detect inflammation and bone destruction in joints earlier than projection radiography (x-ray) in PsA, RA, and spondyloarthritis can [6-8]. US is a tool increasingly used by clinicians, including rheumatologists, but the US data on small joints in PsA are very limited [8,9]; in particular, the validation of the findings is minimal. The aim of the present study was to assess US for the detection of inflammatory and destructive changes in finger and toe joints, tendons, and entheses in patients with PsA by comparison with MRI, x-ray, and clinical findings.

## Materials and methods

### Patients

Fifteen patients with PsA, 5 with RA, and 5 healthy control persons (CTRLs) were examined with US, contrast-enhanced MRI, x-ray, and clinical assessment. The PsA and RA patients were required to have at least one clinically affected finger joint or dactylitis to enter the study. The PsA group included 11 women and 4 men with a median age of 57 years (range 39 to 79) and a median disease duration of 3 years (range 0 to 24). They had a median of 5 tender joints (range 1 to 24) and 2 swollen joints (range 1 to 10). The RA group comprised 5 women with a median age of 48 years (range 32 to 60) and a median disease duration of 7 years (range 0 to 15). Their median tender and swollen joint counts were 8 (range 3 to 9) and 6 (range 2 to 11), respectively. All 5 CTRLs (4 women and 1 man with a median age of 63 years; range 35 to 71) had no prior history of rheumatological disease and no clinically affected joints at inclusion. The study participants signed consent forms after receiving oral and written information. The study was approved by the local Danish ethics committee.

### Ultrasonography

**Figure 1:**
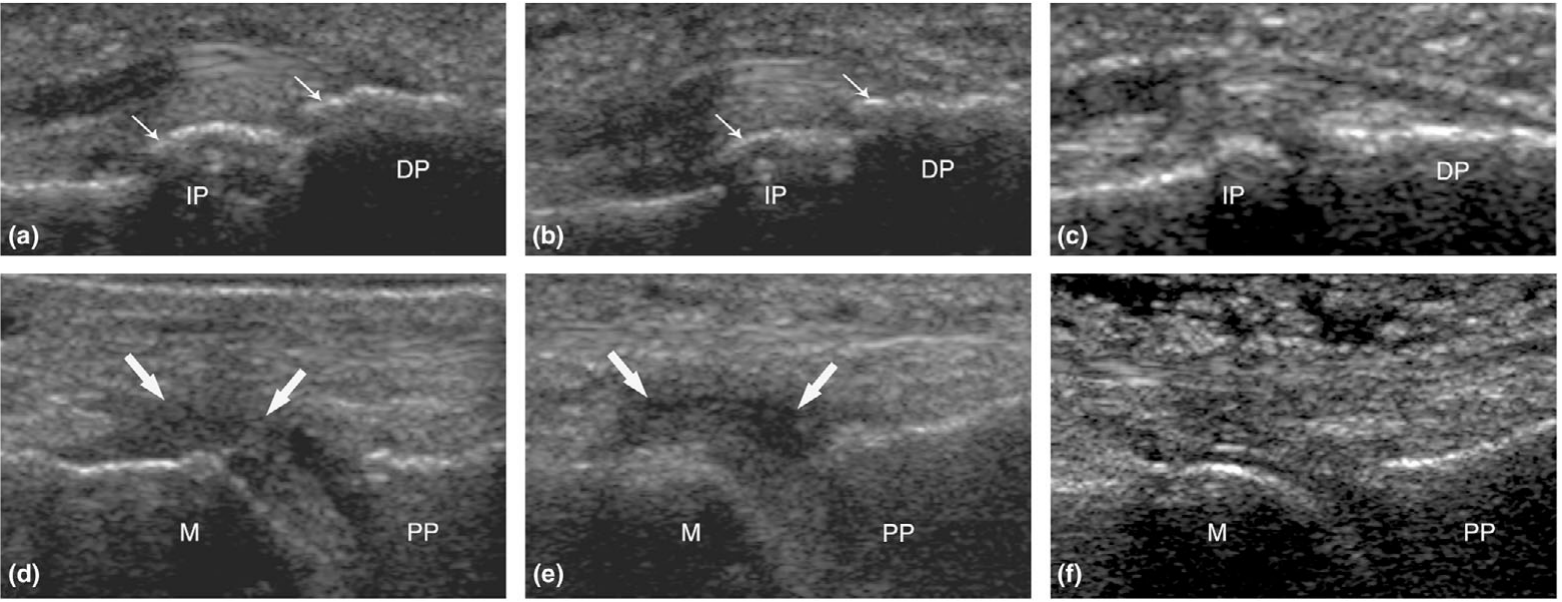
Ultrasonography (US) of distal interphalangeal (DIP) joints **(a-c) **and metatarsophalangeal (MTP) joints **(d-f)**. Images on the left were acquired independently by ultrasonographer 1 (Charlotte Wiell) and middle images were acquired independently by ultrasonographer 2 (Marcin Szkudlarek) in the interobserver US substudy. **(a,b) **Bone proliferations (arrows) in the 2nd DIP joint on US in a palmar view in a patient with psoriasis-associated arthritis (PsA). **(d,e) **Synovitis (arrows) in the 2nd MTP joint on US in a dorsal view in a patient with PsA. Images on the right show a 2nd DIP joint **(c) **and a 2nd MTP joint **(f) **without destructive or inflammatory changes on US. **(f) **Notice subcutaneous edema dorsal to the 2nd MTP joint. DP, distal phalanx; IP, intermediate phalanx; M, metatarsal bone; PP, proximal phalanx.

**Figure 2:**
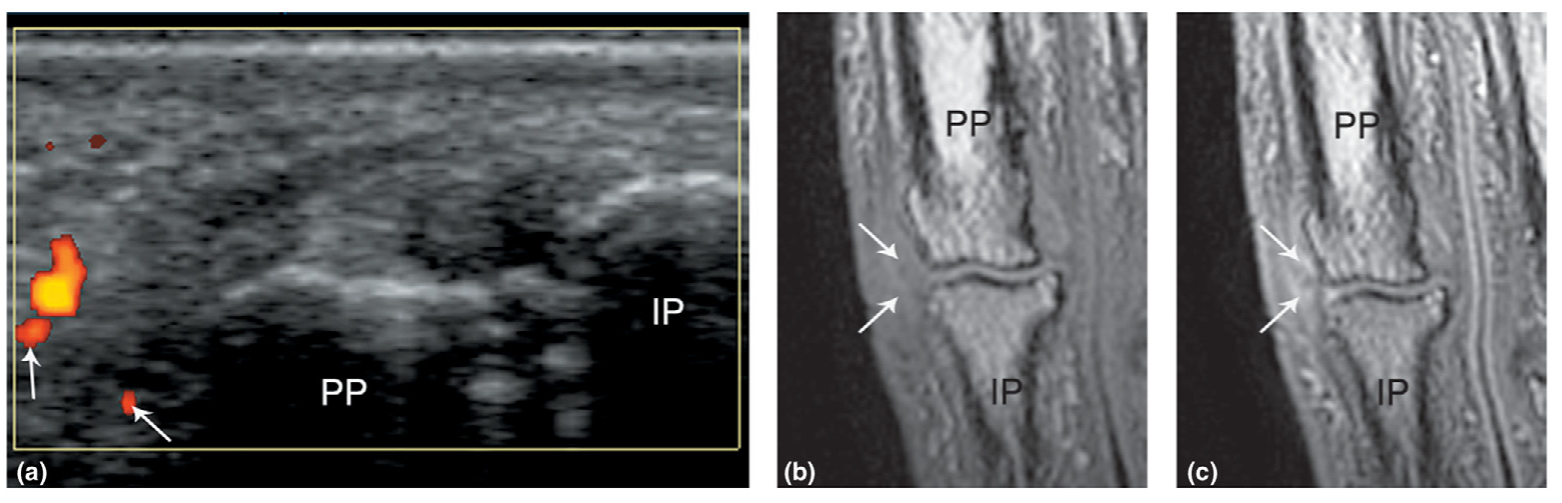
**(a) **Capsular/extracapsular changes (arrows) on power Doppler ultrasonography on the radial side of the 2nd proximal interphalangeal joint in a patient with psoriasis-associated arthritis. **(b,c) **The corresponding coronal T1-weighted magnetic resonance images before **(b) **and after **(c) **contrast administration showing capsular/extracapsular post-contrast enhancement. IP, intermediate phalanx; PP, proximal phalanx.

US was performed with a GE LOGIQ 9 unit (General Electric Medical Systems, now known as GE Healthcare, Little Chalfont, Buckinghamshire, UK) using a high-frequency 9- to 14-MHz linear array transducer. All persons were examined by the same trained ultrasonographer (CW = US1) and examination was repeated in 8 persons (6 PsA, 1 RA, and 1 CTRL) by another trained ultrasonographer (MS = US2), and both US1 and US2 have a rheumatological background (Figure 1). US2 was blinded to diagnosis and clinical data, and both were blinded to other imaging findings, including the sonographic findings of the other ultrasonographer. Bilateral 2nd–5th metacarpophalangeal (MCP), proximal interphalangeal (PIP), and distal interphalangeal (DIP) joints and 1st–5th metatarsophalangeal (MTP) joints were assessed with US for inflammatory changes: synovitis (synovial hypertrophy and/or effusion and/or power Doppler [PD] signal) and capsular/extracapsular PD signal (only in PIP joints) (Figure 2). Furthermore, the tendons of the fingers (2nd–5th flexor and extensor tendons) were assessed for insertional changes (edema and/or calcification and/or periosteal changes and/or PD signal) and tenosynovitis. Finally, all joints were assessed for bone changes: bone erosions and bone proliferations. The presence or absence of each parameter was noted. The palmar and dorsal aspects of each joint were examined in a longitudinal plane. A transverse view was added in case of doubt concerning the type of the detected finding or for confirmation of an erosion. Additional views were radial view of the 2nd MCP joint, ulnar view of the 5th MCP joint, radial and ulnar views of all PIP joints, medial view of the 1st MTP joint, and lateral view of the 5th MTP joint. All views were obtained with the hands and feet in a neutral position. Mild synovitis in joints (score 1 according to the scoring system proposed by Szkudlarek and colleagues [10] for MCP and MTP joints) and a small amount of fluid in the tendon sheath below the flexor tendons at the palmar side of the PIP joints were considered a normal finding. A small amount of fluid around the fat pad on the palmar side of the PIP joint and a synovial membrane thickness below 12 mm (measured at the site of maximal thickness) of the DIP joints were also considered normal (based on unpublished data from CTRLs by Wiell and colleagues). The following US definitions were employed: bone erosion = bone cortex discontinuation in the area adjacent to the joint, visualized in two planes; bone proliferation = bone cortex proliferation in the area adjacent to the joint; synovitis = anechoic or hypoechoic intracapsular area, different from cartilage with or without PD signal; tenosynovitis = hypoechoic rim around the flexor tendon with or without PD signal; capsular/extracapsular changes = PD signal (intracapsular and/or extracapsular at the insertion of capsule or ligament) at the radial or ulnar sides of the PIP joints, different from nutritious vessels; and insertional changes = intratendinous hypoechoic enlargement and/or intratendinous hyperechoic bands with or without acoustic shadow and/or periosteal irregularities and/or intratendinous PD signal at the entheses.

#### Ultrasonography parameters

The setting for grey-scale US was 14 MHz, and the pulse repetition frequency for the PD signal was set at 500 Hz.

### Magnetic resonance imaging

MRI was performed on a Philips Panorama 0.6 tesla unit (Philips Medical Systems, Helsinki, Finland) using a three-channel phased-array solenoid coil within 2 days of the US. The more clinically affected hand (2nd–5th MCP, PIP, and DIP joints) was assessed for the presence or absence of aforementioned changes by a radiologist experienced in musculoskeletal radiography (MH), who was blinded to clinical and other imaging findings.

#### Magnetic resonance imaging parameters

The acquired images included a coronal T1-weighted three-dimensional fast field echo (repetition time [TR] 20 ms, echo time [TE] 8 ms, flip angle 25°, field of view [FOV] 120 mm, matrix 240 × 240, slice thickness [ST] 0.8 mm, number of acquisitions [Acq] 1, and acquisition time [TA] 4.31 minutes) and axial fat saturated T1-weighted (TR 31 ms, TE 11 ms, flip angle 25°, FOV 150 mm, matrix 256 × 256, ST 4 mm, Acq 1, and TA 4.57 minutes) sequences before and after intravenous administration of the contrast agent Omniscan (0.1 mmol/kg; Amersham Health AS, now part of GE Healthcare). Additionally, sagittal (TR 4,000 ms, TE 17 ms, inversion time [TI] 80 ms, flip angle 90°, FOV 160 mm, matrix 256 × 256, ST 3 mm, Acq 1, and TA 6.56 minutes) and axial (TR 3,000 ms, TE 17 ms, TI 80 ms, flip angle 90°, FOV 160 mm, matrix 256 × 256, ST 3 mm, Acq 1, and TA 7.01 minutes) short tau inversion recovery (STIR) sequences were performed before contrast administration. Reconstructions were performed with a ST that was half of the acquired ST.

### Projection radiography

X-ray of hands and feet in a posterior-anterior projection was performed within a month of the US. X-rays of both hands and feet (2nd–5th MCP, PIP, DIP, and MTP joints) were assessed for bone erosions and bone proliferations according to the Ratingen scoring system [11] by an experienced musculoskeletal radiologist (AV) blinded to clinical and other imaging findings.

### Clinical examination

All 25 persons underwent clinical examination prior to US to determine the presence or absence of swelling and/or tenderness of the finger and MTP joints (in all 34 joints per person). One patient with PsA had had joint replacement in 4 MCP joints and total anchylosis in one PIP joint. These joints were not assessed.

### Statistical analysis

Absolute agreements and unweighted kappa values between US (US1), MRI, x-ray, and clinical examination were calculated. Furthermore, the interobserver agreement between US1 and US2 was determined. Kappa values below 0.20 were considered poor, 0.21 to 0.40 fair, 0.41 to 0.60 moderate, 0.61 to 0.80 good, and 0.81 to 1.00 very good [12]. MRI was used as the standard reference method for the calculation of the sensitivity and specificity of US, x-ray, and clinical examination [12]. The statistical software used was SPSS version 12.0 for Windows (SPSS Inc., Chicago, IL, USA).

## Results

A total of 845 joints were examined by US, 300 by MRI, and 795 by x-ray.

### Ultrasonography observations in PsA and RA patients and CTRLs

**Figure 3:**
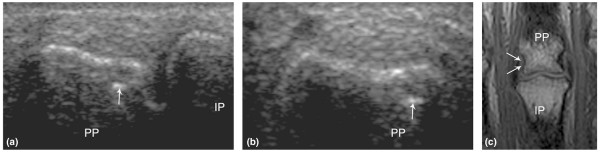
**(a) **Bone cortex defect (arrows) on ultrasonography in a 63-year-old healthy control person on the radial side of the 3rd proximal interphalangeal joint in a longitudinal view. **(b) **The corresponding transverse view. **(c) **The coronal T1-weighted magnetic resonance image without contrast administration reveals no erosion-like changes in the same person at the corresponding site (arrows). IP, intermediate phalanx; PP, proximal phalanx.

The observations by US in PsA and RA patients and CTRLs are listed in Table 1. In particular, it was noted that the DIP joints of patients with PsA had more pathological findings than RA patients; especially, no bone changes (erosions and proliferations) were present in the RA patients. Bone changes found in the MTP joints were primarily at the medial side of the 1st MTP joint. In CTRLs, nine erosion-like changes were seen by US (Figure 3). All were located at the radial and ulnar side of the 2nd and 3rd PIP joints and the 5th MCP joints and at the medial side of 1st MTP joints. In the finger joints, the size of the bone cortex defect was below 12 mm; in the MTP joints, the size was below 20 mm. Bone proliferations were found in two CTRL DIP joints. Synovitis was common in both groups of patients, although we found a tendency toward more synovitis in the MCP and PIP joints in RA patients. A high frequency of synovitis was registered in MTP joints, including in CTRLs. The frequency of tenosynovitis was generally higher in RA than PsA patients, whereas insertional changes and capsular/extracapsular changes were found more frequently, but not exclusively, in PsA.

**Table 1:** Ultrasonography observations in psoriasis-associated arthritis and rheumatoid arthritis patients and healthy control persons

	All	Psoriasis-associated arthritis	Rheumatoid arthritis	Healthy control persons
Bone erosions				
MCP joint	12%	13%	18%	3%
PIP joint	12%	14%	3%	13%
DIP joint	3%	4%	0%	0%
MTP joint	15%	15%	24%	6%
Bone proliferations				
MCP joint	4%	6%	0%	0%
PIP joint	8%	12%	3%	0%
DIP joint	9%	13%	0%	5%
MTP joint	5%	5%	4%	6%
Synovitis				
MCP joint	22%	19%	50%	3%
PIP joint	13%	13%	23%	3%
DIP joint	18%	22%	23%	3%
MTP joint	44%	43%	56%	34%
Tenosynovitis				
MCP joint	7%	4%	23%	0%
PIP joint	18%	16%	40%	0%
DIP joint	6%	2%	20%	3%
Insertional changes				
Extensor tendons	8%	12%	3%	3%
Flexor tendons	8%	7%	18%	0%
Capsular/extracapsular changes	9%	7%	25%	0%

Two hundred fingers (196 MCP, 199 PIP, and 200 DIP joints) and 250 MTP joints were examined. The distribution was as follows: psoriasis-associated arthritis = 60 fingers (56 MCP, 59 PIP, and 60 DIP joints) and 150 MTP joints; rheumatoid arthritis = 20 fingers (20 MCP, 20 PIP, and 20 DIP joints) and 50 MTP joints; and healthy control persons = 20 fingers (20 MCP, 20 PIP, and 20 DIP joints) and 50 MTP joints. DIP, distal interphalangeal; MCP, metacarpophalangeal; MTP, metatarsophalangeal; PIP, proximal interphalangeal.

### Observations by ultrasonography, MRI, and x-ray in the MRI-examined hand

The observations by US, MRI, and x-ray in the MRI-examined hand are listed in Table 2. Both US and MRI were more sensitive in detecting bone changes than x-ray, except in DIP joints. US and MRI found inflammatory changes with an equal frequency, although US discovered slightly more than MRI in the distal part of the finger. In particular, it was noted that US found more erosions in the PIP joints than either MRI or x-ray. The opposite was the case for DIP joint erosions. US and MRI detected more bone proliferations in the MCP and PIP joints and more erosions in the MCP joints than x-ray. US generally detected pathological changes (except erosions) in the DIP joints more frequently than MRI. None of the bone changes (four erosion-like changes) found by US in CTRLs was confirmed by MRI. Synovitis was not registered by US in any CTRL and was found in only one PIP joint by MRI. Synovitis was discovered more frequently in the DIP joint by US, whereas no apparent difference between US and MRI was found in the other finger joints. Insertional changes were seen with a comparable frequency by US and MRI, although more changes at the insertion of the flexor tendons were registered by US. Capsular/extracapsular changes were predominantly visualized by US.

**Table 2:** Ultrasonography, MRI, and x-ray findings in the MRI-examined hand

	Ultrasonography	MRI	X-ray
Bone erosions			
MCP joint (total)	15%	16%	7%
PsA	18%	23%	12%
RA	15%	10%	0%
PIP joint (total)	15%	7%	5%
PsA	20%	8%	7%
RA	0%	10%	5%
DIP joint (total)	1%	3%	5%
PsA	2%	5%	8%
RA	0%	0%	0%
Bone proliferations			
MCP joint (total)	4%	3%	0%
PsA	7%	5%	0%
RA	0%	0%	0%
PIP joint (total)	7%	6%	0%
PsA	12%	10%	0%
RA	0%	0%	0%
DIP joint (total)	7%	2%	4%
PsA	12%	3%	7%
RA	0%	0%	0%
Synovitis			
MCP joint (total)	28%	27%	NA
PsA	28%	35%	NA
RA	55%	30%	NA
PIP joint (total)	22%	20%	NA
PsA	27%	23%	NA
RA	30%	25%	NA
DIP joint (total)	12%	5%	NA
PsA	18%	7%	NA
RA	5 %	5%	NA
Tenosynovitis			
MCP joint (total)	6%	13%	NA
PsA	2%	12%	NA
RA	25%	30%	NA
PIP joint (total)	20%	7%	NA
PsA	18%	5%	NA
RA	45%	20%	NA
DIP joint (total)	6%	6%	NA
PsA	2%	3%	NA
RA	25%	20%	NA
Insertional changes			
Extensor tendons (total)	6%	4%	NA
PsA	8%	7%	NA
RA	5%	0%	NA
Flexor tendons (total)	9%	4%	NA
PsA	3%	0%	NA
RA	35%	20%	NA
Capsular/extracapsular changes (total)	18%	7%	NA
PsA	22%	7%	NA
RA	25%	15%	NA

One hundred fingers (100 MCP, 100, PIP, and 100 DIP joints) ('total') were examined. The distribution was as follows: psoriasis-associated arthritis (PsA) = 60; rheumatoid arthritis (RA) = 20; and healthy control persons (not shown) = 20. The imaging modalities were ultrasonography, magnetic resonance imaging (MRI), and projection radiography (x-ray). DIP, distal interphalangeal; MCP, metacarpophalangeal; NA, not applicable; PIP, proximal interphalangeal.

### Ultrasonography interobserver agreement

The results of the US interobserver substudy are listed in Table 3 (bone changes) and Table 4 (inflammatory changes). The absolute agreements for both bone and inflammatory changes were high (median 96%; range 50% to 100%), except for synovitis and tenosynovitis at the PIP joints in patients with RA (50% and 63%). The kappa values were good to very good for all bone changes (kappa 0.60 to 1.00), except for bone proliferations in MTP joints (0.52) and erosions in PIP joints (0.52). The strength of agreement for synovitis in MTP joints was good to very good (kappa 0.78 to 1.00) but for other inflammatory findings the agreements were poor to fair (kappa -0.05 to 0.37). US1 registered pathological findings more frequently than US2.

**Table 3:** Agreements between US, MRI, and x-ray for bone changes

	US1 versus US2	US versus MRI	US versus x-ray	MRI versus x-ray
Bone erosions				
MCP joint (total)	95%; κ = 0.795	87%; κ = 0.504	89%; κ = 0.268	89%; κ = 0.470
PsA	95%; κ = 0.830	85%; κ = 0.547	88%; κ = 0.358	85%; κ = 0.492
RA	88%; κ = 0.600	85%; κ = 0.318	83%; κ = NA	90%; κ = NA
CTRL	100%; κ = NA	95%; κ = NA	95%; κ = NA	100%; κ = NA
PIP joint (total)	86%; κ = 0.522	86%; κ = 0.296	87%; κ = 0.361	92%; κ = 0.292
PsA	83%; κ = 0.526	85%; κ = 0.400	85%; κ = 0.411	88%; κ = 0.160
RA	100%; κ = NA	90%; κ = NA	93%; κ = 0.375	95%; κ = 0.643
CTRL	88%; κ = NA	85%; κ = NA	88%; κ = NA	100%; κ = NA
DIP joint (total)	100%; κ = 1.000	96%; κ = -0.015	96%; κ = 0.506	96%; κ = 0.481
PsA	100%; κ = 1.000	93%; κ = -0.026	93%; κ = 0.527	93%; κ = 0.467
RA	100%; κ = NA	100%; κ = NA	100%; κ = NA	100%; κ = NA
CTRL	100%; κ = NA	100%; κ = NA	100%; κ = NA	100%; κ = NA
MTP joint (total)	90%; κ = 0.702	-	89%; κ = 0.259	-
PsA	90%; κ = 0.799	-	89%; κ = 0.378	-
RA	100%; κ = 1.000	-	78%; κ = -0.084	-
CTRL	90%; κ = 0.783	-	100%; κ = NA	-
Bone proliferations				
MCP joint (total)	97%; κ = 0.783	93%; κ = -0.036	96%; κ = NA	97%; κ = NA
PsA	95%; κ = 0.776	88%; κ = -0.061	94%; κ = NA	95%; κ = NA
RA	100%; κ = NA	100%; κ = NA	100%; κ = NA	100%; κ = NA
CTRL	100%; κ = NA	100%; κ = NA	100%; κ = NA	100%; κ = NA
PIP joint (total)	92%; κ = 0.665	93%; κ = 0.424	93%; κ = 0.117	94%; κ = NA
PsA	89%; κ = 0.648	88%; κ = 0.397	89%; κ = 0.120	90%; κ = NA
RA	100%; κ = NA	100%; κ = NA	98%; κ = NA	100%; κ = NA
CTRL	100%; κ = NA	100%; κ = NA	100%; κ = NA	100%; κ = NA
DIP joint (total)	95%; κ = 0.851	95%; κ = 0.427	94%; κ = 0.472	98%; κ = 0.658
PsA	94%; κ = 0.838	92%; κ = 0.414	91%; κ = 0.476	97%; κ = 0.651
RA	100%; κ = NA	100%; κ = NA	100%; κ = NA	100%; κ = NA
CTRL	100%; κ = NA	100%; κ = NA	95%; κ = NA	100%; κ = NA
MTP joint (total)	93%; κ = 0.515	-	99%; κ = NA	-
PsA	92%; κ = 0.250	-	98%; κ = NA	-
RA	100%; κ = 1.000	-	100%; κ = NA	-
CTRL	100%; κ = NA	-	100%; κ = NA	-

Values are absolute agreement presented as a percentage. The imaging modalities were ultrasonography (US), magnetic resonance imaging (MRI), and projection radiography (x-ray). The interobserver substudy was performed by ultrasonographer 1 (Charlotte Wiell = US1) and ultrasonographer 2 (Marcin Szkudlarek = US2). A hyphen (-) indicates that the measurement was not performed. κ, kappa value; CTRL, healthy control person; DIP, distal interphalangeal; MCP, metacarpophalangeal; MTP, metatarsophalangeal; NA, not applicable; PIP, proximal interphalangeal; PsA, psoriasis-associated arthritis; RA, rheumatoid arthritis.

**Table 4:** Agreements between US, MRI, and clinical examination for inflammatory changes

	US1 versus US2	US versus MRI	US^a ^versus clinical examination^b^	MRI^c ^versus clinical examination^b^
Synovitis				
MCP joint (total)	88%; κ = 0.305	83%; κ = 0.574	62%; κ = -0.033	63%; κ = 0.047
PsA	84%; κ = 0.280	80%; κ = 0.540	62%; κ = 0.047	64%; κ = 0.131
RA	100%; κ = NA	75%; κ = 0.519	63%; κ = -0.007	60%; κ = 0.091
CTRL	100%; κ = NA	100%; κ = NA	63%; κ = NA	100%; κ = NA
PIP joint (total)	78%; κ = 0.361	78%; κ = 0.337	66%; κ = NA	66%; κ = 0.012
PsA	84%; κ = 0.367	73%; κ = 0.290	64%; κ = -0.053	68%; κ = 0.149
RA	50%; κ = 0.059	75%; κ = 0.375	63%; κ = 0.162	50%; κ = -0.190
CTRL	100%; κ = NA	95%; κ = NA	73%; κ = NA	95%; κ = NA
DIP joint (total)	83%; κ = 0.129	87%; κ = 0.177	74%; κ = -0.020	79%; κ = -0.082
PsA	84%; κ = 0.120	78%; κ = 0.039	72%; κ = 0.041	68%; κ = -0.098
RA	100%; κ = NA	100%; κ = 1.000	83%; κ = -0.077	90%; κ = -0.053
CTRL	100%; κ = NA	100%; κ = NA	73%; κ = NA	100%; κ = NA
MTP joint (total)	91%; κ = 0.825	-	51%; κ = -0.063	-
PsA	90%; κ = 0.799	-	55%; κ = -0.038	-
RA	100%; κ = 1.000	-	52%; κ = -0.034	-
CTRL	90%; κ = 0.783	-	38%; κ = NA	-
Tenosynovitis				
MCP joint (total)	93%; κ = -0.034	91%; κ = 0.484	-	-
PsA	91%; κ = -0.048	90%; κ = 0.227	-	-
RA	100%; κ = NA	85%; κ = 0.625	-	-
CTRL	100%; κ = NA	100%; κ = NA	-	-
PIP joint (total)	87%; κ = -0.029	96%; κ = 0.645	-	-
PsA	89%; κ = -0.035	87%; κ = 0.380	-	-
RA	63%; κ = NA	75%; κ = 0.468	-	-
CTRL	100%; κ = NA	100%; κ = NA	-	-
DIP joint (total)	97%; κ = NA	96%; κ = 0.645	-	-
PsA	98%; κ = NA	98%; κ = 0.659	-	-
RA	88%; κ = NA	85%; κ = 0.571	-	-
CTRL	100%; κ = NA	100%; κ = NA	-	-
Insertional changes				
Extensor tendons (total)	88%; κ = NA	94%; κ = 0.370	-	-
PsA	83%; κ = NA	92%; κ = 0.400	-	-
RA	100%; κ = NA	95%; κ = NA	-	-
CTRL	100%; κ = NA	100%; κ = NA	-	-
Flexor tendons (total)	98%; κ = NA	95%; κ = 0.593	-	-
PsA	98%; κ = NA	97%; κ = NA	-	-
RA	100%; κ = NA	85%; κ = 0.634	-	-
CTRL	100%; κ = NA	100%; κ = NA	-	-
Capsular/extra-capsular changes				
Total	86%; κ = NA	87%; κ = 0.511	-	-
PsA	83%; κ = NA	88%; κ = 0.410	-	-
RA	88%; κ = NA	90%; κ = 0.692	-	-
CTRL	100%; κ = NA	100%; κ = NA	-	-

^a^Ultrasonography (US) synovitis (synovial hypertrophy and/or effusion, and/or power Doppler signal); ^b^clinically tender and swollen joints; ^c^magnetic resonance imaging (MRI) synovitis. Values are absolute agreement presented as a percentage. The imaging modalities were US, MRI, and clinical examination. The interobserver substudy was performed by ultrasonographer 1 (Charlotte Wiell = US1) and ultrasonographer 2 (Marcin Szkudlarek = US2). A hyphen (-) indicates that the measurement was not performed. κ, kappa value; CTRL, healthy control person; DIP, distal interphalangeal; MCP, metacarpophalangeal; MTP, metatarsophalangeal; NA, not applicable; PIP, proximal interphalangeal; PsA, psoriasis-associated arthritis; RA, rheumatoid arthritis.

### Agreement between ultrasonography, MRI, x-ray, and clinical examination

**Figure 4:**
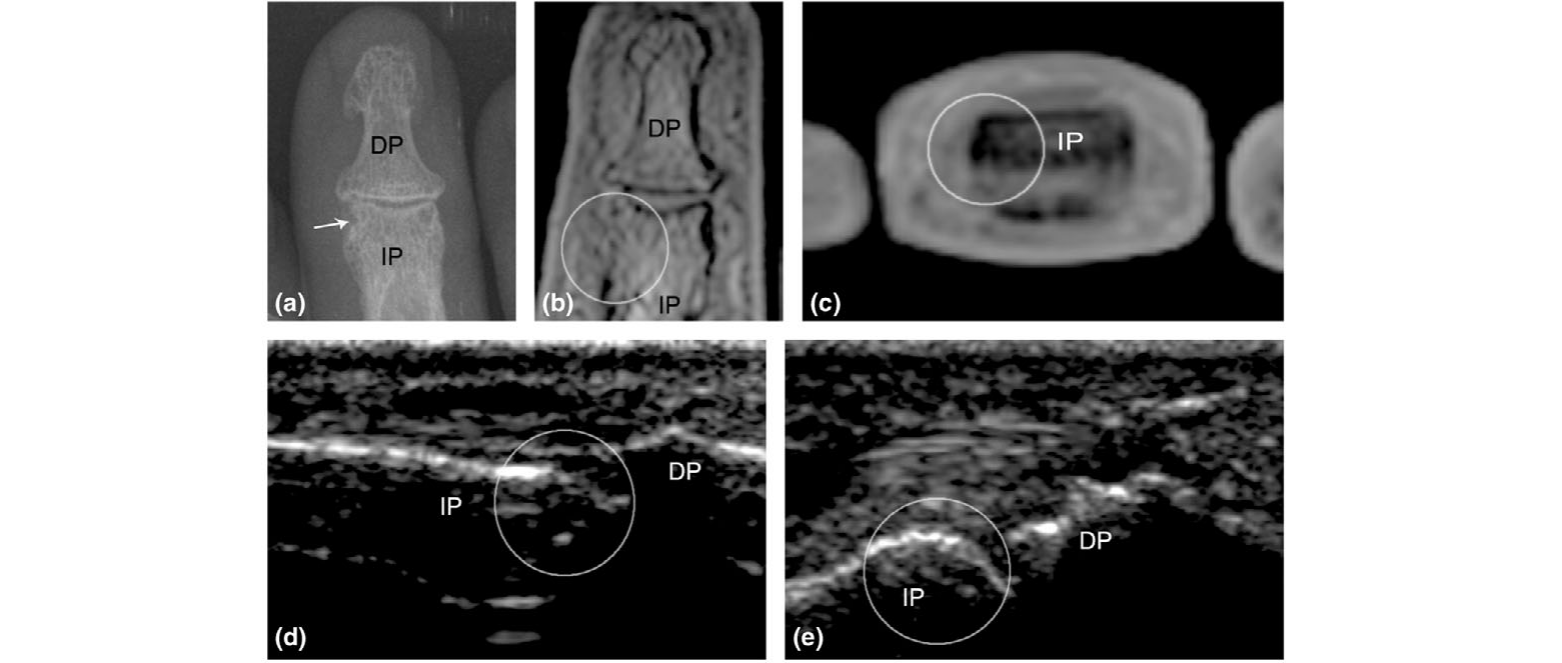
Projection radiography (x-ray) **(a) **and T1-weighted coronal **(b) **and axial **(c) **magnetic resonance imaging (MRI) and ultrasonography (US) in longitudinal dorsal **(d) **and palmar **(e) **views of the 3rd distal interphalangeal joint of a patient with psoriasis-associated arthritis. An erosion was scored on x-ray **(a) **but not on either MRI **(b,c) **or US **(d,e)**, even though small irregularities were seen. DP, distal phalanx; IP, intermediate phalanx.

The agreements between US, MRI, and x-ray for bone changes are listed in Table 3, and the agreements between US, MRI, and clinical examination for inflammatory changes are listed in Table 4. The absolute agreement between the imaging modalities was generally high for bone changes (median 95%; range 78% to 100%) and was lowest for erosions in MTP joints. The absolute agreements between US and MRI for inflammatory changes were slightly lower (median 91%; range 73% to 100%) and were lowest for synovitis and tenosynovitis in the PIP joints. In contrast, the kappa values were higher for inflammatory changes (median 0.51; range 0.04 to 1.00) than for bone changes (median 0.40; range -0.08 to 0.66). X-ray findings that were not revealed by US included erosions in 3 MCP joints (PsA), 13 PIP joints (10 PsA and 3 RA), and 8 DIP joints (PsA) and proliferations in 3 DIP joints (PsA). In the 2nd–4th MTP joints, x-ray detected 6 erosions that were not found by US (4 PsA and 2 RA). Most of the erosions visible by x-ray and not by US were in the non-MRI-examined hand. However, 3 x-ray erosions (all in PsA: 1 MCP and 2 DIP joints) and 2 proliferations (both PsA: 2 DIP joints) were located in the MRI-examined hand and were not registered by MRI (Figure 4). In 2 of the erosions, the anatomy was not fully covered. US versus clinical examination agreements and MRI versus clinical examination agreements were generally low (median 65%; range 38% to 100%) and were lowest for MTP joints. Generally, agreements on the absence of findings were more frequent than agreements on the presence of findings.

### Sensitivities and specificities of ultrasonography, x-ray, and clinical examination, with MRI as standard reference method

Sensitivities and specificities of US, x-ray, and clinical examination, with MRI as the standard reference method, are listed in Table 5. The specificity of US and x-ray was high for all pathologies. It is noted that US was more sensitive than x-ray for detecting erosions, except in DIP joints. The sensitivity for bone proliferations in DIP joints was high for both US and x-ray. The sensitivity of US was highest for synovitis in MCP joints and for tenosynovitis in PIP joints. The sensitivity of US for detecting insertional changes and capsular/extracapsular changes was high, except for insertional changes in the extensor tendons. US consistently found more joints with synovitis than the corresponding clinical examination (median sensitivity 0.50 versus 0.25).

**Table 5:** Sensitivity and specificity of US, x-ray, and clinical examination, with MRI as the standard reference method

	US sensitivity	US specificity	X-ray sensitivity	X-ray specificity	Clinical examination sensitivity	Clinical examination specificity
Bone erosions						
MCP joint	0.56	0.93	0.38	0.99	NA	NA
PIP joint	0.57	0.88	0.40	0.95	NA	NA
DIP joint	0.00	0.99	0.67	0.97	NA	NA
Bone proliferations						
MCP joint	0.00	0.97	0.00	0.96	NA	NA
PIP joint	0.50	0.96	0.00	0.96	NA	NA
DIP joint	1.00	0.95	1.00	0.98	NA	NA
Synovitis						
MCP joint	0.70	0.88	NA	NA	0.31	0.74
PIP joint	0.50	0.88	NA	NA	0.25	0.76
DIP joint	0.40	0.87	NA	NA	0.00	0.83
Tenosynovitis						
MCP joint	0.38	0.99	NA	NA	NA	NA
PIP joint	1.00	0.86	NA	NA	NA	NA
DIP joint	0.67	0.98	NA	NA	NA	NA
Insertional changes						
Extensor tendons	0.50	0.96	NA	NA	NA	NA
Flexor tendons	1.00	0.95	NA	NA	NA	NA
Capsular/extracapsular changes	1.00	0.88	NA	NA	NA	NA

The imaging modalities were ultrasonography (US), magnetic resonance imaging (MRI), projection radiography (x-ray), and clinical examination. DIP, distal interphalangeal; MCP, metacarpophalangeal; NA, not applicable; PIP, proximal interphalangeal.

## Discussion

To our knowledge, this is the first study to examine small joints in PsA using high-end US and comparing it with contrast-enhanced MRI, x-ray, and clinical examination. A higher frequency of DIP joint changes was found by US in the PsA patients compared with RA patients. In particular, DIP joint bone changes were found exclusively in PsA. Furthermore, bone proliferations were more common and tenosynovitis was less frequent in PsA than RA. For other pathologies, no disease-specific pattern was observed. US and MRI were more sensitive to inflammatory and destructive changes than x-ray and clinical examination, and US showed a high interobserver agreement for bone changes and a lower interobserver agreement for inflammatory changes (Figure 1). A high absolute agreement (85% to 100%) for all destructive changes and a more moderate absolute agreement (73% to 100%) for the inflammatory pathologies were found between US and MRI.

In this study, US revealed synovitis more frequently in MCP and PIP joints and bone erosions less frequently in PIP joints in the RA group than in the PsA group, whereas Fournié and colleagues [13] reported minimal differences in the amount of erosion and synovitis in MCP and PIP joints of PsA and RA patients. Fournié and colleagues [13], as in our study, reported more tenosynovitis and a few osteoarthritic changes (2 of 21 patients) in the RA group and only erosive DIP joint changes in the PsA group. However, they exclusively found extrasynovial changes in PsA patients, which we also detected in 3 of the 5 RA patients (60% confirmed by MRI). Larger studies are required to provide final conclusions. Erosion-like changes were detected by US in 5% of the CTRL joints (6 in fingers and 3 in MTP joints) in our study (Figure 3). Szkudlarek and colleagues found erosion-like changes in 1% of the MTP joints [14] and 0% of the finger joints (MCP and PIP) [15], whereas Døhn and colleagues [7] found erosion-like changes in 38% (6 of 16) and Wakefield and colleagues [16] in 1% (1 of 100) of the examined MCP joints. Five of the 6 erosion-like changes found in our study were located at the radial or ulnar side of the 2nd and 3rd PIP joints and were all very small. Schmidt and colleagues [17] did a circumferential scan of the 2nd PIP joints on 102 CTRLs and reported no erosive changes. Døhn and colleagues [7] suggested that US may be too sensitive, as MRI and computed tomography (CT) could not confirm any of the erosion-like changes found by US. Similarly, none of the US erosion-like changes in our study was confirmed by MRI. The changes may be explained by a high US sensitivity, but some may be physiologic bone notches mistaken for erosions. Future studies with CT as the standard reference method or longitudinal prognostic US studies can provide stronger evidence on bone changes. Both synovitis, especially in MTP joints [14,15], and tenosynovitis [17,18] have been reported in CTRLs. Szkudlarek and colleagues [14,15] reported synovitis in 8% of MTP joints compared with 34% in our study and 2.5% in MCP and PIP joints compared with our 6%. Schmidt and colleagues [17] found signs of tenosynovitis, defined as a hypoechoic rim around 97% of the 2nd flexor tendons, whereas we detected this in only 3%. However, in advance, we excluded minimal changes in our calculations (Materials and methods). The fact that both US1 and US2 found a high frequency of MTP joint synovitis indicates that this observation was truly frequent in our control population. Our high frequency of MTP synovitis in CTRLs may be partly caused by asymptomatic osteoarthritis (OA), as one third are found in the 1st MTP joint, which is often involved in OA. Another contributing cause may be that the applied definitions of synovitis were too sensitive. However, the definitions of MTP synovitis suggested by Koski and colleagues [19] and Schmidt and colleagues [17] also included some of the cases of MTP synovitis in CTRLs, which were found using our definitions. Our results were obtained using US from dorsal, palmar/plantar, and lateral projections. Further studies are needed to determine whether examination from one or more planes can be omitted without marked loss of sensitivity.

In the present study, US and MRI were more sensitive than x-ray and clinical examination in both PsA and RA, which is in agreement with previous findings in RA [7-9,14-16,18,20,21]. It is of major interest whether bone changes, especially proliferations, found in DIP joints in PsA can be distinguished from bone changes found in OA. This has been addressed by Tan and colleagues [22], who reported that DIP joints on MRI more frequently showed enthesitis enhancement, entheseal erosion, extracapsular changes, and diffuse bone edema in PsA than in OA. We did not examine an OA group, but bone proliferations and insertional changes found in DIP joints of two 63-year-old CTRLs were probably caused by asymptomatic OA. Furthermore, all bone proliferations found in CTRL MTP joints were located on the medial side of the 1st MTP joint, which is a frequent location of OA. The same was the case in the RA group, suggesting concomitant OA as the cause, whereas only three of the seven proliferations found in the PsA group were located there.

US is often criticized for being operator-dependent. Our analysis of interobserver agreement showed a higher agreement for both inflammatory and destructive changes (median 96%) than seen in a European League Against Rheumatism (EULAR) interobserver study [23] in which 14 experienced ultrasonographers examined fingers and wrist (median 73%). The pattern of higher kappa values for bone changes than for inflammatory changes found in our study is concordant with other studies [10,23].

US and MRI showed high concordance (85% to 100%) for all destructive changes and a more moderate concordance (73% to 100%) for the inflammatory pathologies. This lower agreement for inflammatory changes has also been reported by others [8,14,15]. Szkudlarek and colleagues [15] found an overall agreement for synovitis of 76% (versus 82% in our study). In the EULAR study, the overall agreement between US and MRI for fingers and wrist was 73% [23]. The agreement between US and MRI for tenosynovitis was low. Contributing causes may be that US can detect small tendon sheath effusions (especially at the PIP joints) that may not show post-contrast enhancement on MRI [24] and that it can be difficult to distinguish synovitis from tenosynovitis by US. The latter very probably also contributes to the rather low US interobserver reproducibility for synovitis and tenosynovitis.

X-ray is the routine imaging modality for following the destructive changes in clinical practice in patients with arthritis. Many RA studies have shown higher sensitivity of US than x-ray for detecting erosions in RA patients [15,16,21,24], without loss of specificity [15]. Our US sensitivities for erosions, when MRI was considered the standard reference method, showed the same tendency, although not as clearly as in RA [15]. A contributing cause to the lower sensitivity in our study could be that we examined smaller anatomical structures (DIP joints and entheses included). Also, on MRI, detecting such changes may be difficult. This explains why x-ray found some bone changes that were not revealed by US and MRI and why US detected some pathologies (small bone proliferations, DIP synovitis, and insertional changes) more frequently than MRI. In particular, the distinction of capsular enhancement from extracapsular enhancement was difficult by MRI. However, this was met by scoring the two together. The present results were obtained with a 0.6-tesla magnet, and MRI data that are even more detailed may be obtained with higher field strengths (1.5-tesla or 3.0-tesla MRI). As findings by MRI are minimally validated, it can be debated whether MRI is the ideal standard reference. CT, though not better validated than MRI, is a potentially superior option as a reference method for bone changes. Clinical examination showed lower sensitivity (median 0.25) than US (median 0.50) for the detection of inflammatory changes (synovitis). This is in agreement with several previous reports [8,14,15,18].

## Conclusion

The present study has demonstrated that both US and MRI are more sensitive for visualization of inflammatory and destructive changes in fingers and toes of patients with PsA. The US interobserver agreement was high for bone changes but was lower for inflammatory changes, whereas the intermodality (US versus MRI) agreement was moderate to high. In comparison with RA patients, PsA patients showed more DIP joint changes. Furthermore, bone proliferations were more common and tenosynovitis was less frequent in PsA than RA. Even though further studies are needed (for example, on definitions of pathologies, standardization of methods, sensitivity to change, and prognostic value), it seems evident that both US and MRI have major potential for improved examination of joints, tendons, and entheses in fingers and toes of patients with PsA.

## Abbreviations

Acq = number of acquisitions; CT = computed tomography; CTRL = healthy control person; DIP = distal interphalangeal; EULAR = European League Against Rheumatism; FOV = field of view; MCP = metacarpophalangeal; MRI = magnetic resonance imaging; MTP = metatarsophalangeal; OA = osteoarthritis; PD = power Doppler; PIP = proximal interphalangeal; PsA = psoriasis-associated arthritis; RA = rheumatoid arthritis; ST = slice thickness; TA = acquisition time; TE = echo time; TI = inversion time; TR = repetition time; US = ultrasonography; US1 = ultrasonographer 1 (Charlotte Wiell); US2 = ultrasonographer 2 (Marcin Szkudlarek); x-ray = projection radiography.

## Competing interests

The authors declare that they have no competing interests.

## Authors' contributions

CW participated in the study development and recruitment of patients, performed the ultrasonographic examinations (US1), conducted data evaluation and statistical analysis, and prepared the manuscript draft. MS participated in the study development, performed ultrasonographic examinations (US2), and gave substantial input to data evaluation and manuscript preparation. MH was involved in the MRI scanning protocol and evaluated MRI images. JMM was involved in the MRI scanning protocol and performed all MRI examinations. AV evaluated the radiographs. JN participated in the study development and gave substantial input to the data evaluation and manuscript preparation. LT gave substantial input to the data evaluation and manuscript preparation. MØ participated in the study development, was involved in the MRI scanning protocol, and gave substantial input to the data evaluation and manuscript preparation. All authors read and approved the final manuscript.
